# A Case of Rheumatic Suppuration, Successfully Treated

**Published:** 1807-03

**Authors:** John W. Montross

**Affiliations:** Montgomery, Orange County, America


*A* CASE *of* RHEUMATIC SUPPURATION, *successfully treated. By* DOCTOR JOHN W. MONTROSS, *of Montgomery, Orange County, America.*

IN the latter part of October of the year 1803, I took a man under my care, who had been confined to his bed for about the space of six months, with a disease in one of his thighs, answering to the description given by Mr. Bell of a white swelling of the rheumatic kind. The disease had been in its suppurative stage, I think, at least three months, and discharged, I suppose, betwixt one and two pounds of pus per day.

The man was reduced to the most pitiable state, and his life despaired of. My manner of treatment was, to exhibit about one and a half, or two grains of calomel per day, and as much opium as was requisite to relieve him of pain, which was extremely acute. This method of treatment was continued till a copious ptyalism was indnced; I then desisted from the use of calomel, and continued the opium alone, as occasion required. A strict bandage was applied the whole length of his thigh, and an incision, at his knee, kept open with a tent, through which a probe could be run, betwixt six and eight inches up the muscles, in a cavity of considerable latitude. From the time the ptyalism commenced, the discharge from the ulcer began to decline, and in a short time entirely ceased, and the cavity healed. The man now began to regain his flesh at an astonishing rate; and appearing to be in good health, the use of opium was now now gradually left off; but, in a short time, he had severe pains in his arms and in the diseased thigh, which again began to tumefy. I again resorted to the exhibition of calomel, and the application of blue mercurial ointment to his thigh. The calomel was continued till a slight ptyalism was induced, which immediately relieved him of pain. This was followed by the exhibition of tonics, for about two or three weeks; and the man has enjoyed from that time perpetual health.

It may be proper further to relate of this case, that a large callous tumour extended round the articulation, and that the limb could not be brought within forty degrees of a straight line, and that the tumour and crookedness continued undiminished after the ulcer was cured.

By my direction he continued, through the winter, to rub the tumour with blue mercurial ointment, which, by spring, resolved the tumour, and restored the limb to its former straightness. My principal motive for communicating the above is, that Mr. Bell has spoken so discouragingly of the cure of the disease, after it arrives at a state of suppuration, and recommends mercury for its cure, but not in this stage of it; which, to those practitioners who implicitly follow authors, is a very embarrassing circumstance.

One circumstance, however, occurs to me, which may not be altogether unimportant to mention. Some time in July I went with the physician who then attended him, and on examining his thigh, we both concluded that there was pus formed a little above the joint of the knee. He made an incision through about three quarters of an inch of flesh, and came to a cavity which contained no matter, and in which he could pass his probe an inch or two around, and in a direction upwards, I think, near three inches.

*New York, April* 4, 1805.

